# Can small molecular inhibitors that stop de novo serine synthesis be used in cancer treatment?

**DOI:** 10.1038/s41420-021-00474-4

**Published:** 2021-04-30

**Authors:** Megan Jessica McNamee, David Michod, Maria Victoria Niklison-Chirou

**Affiliations:** 1 Centre for Therapeutic Innovation (CTI-Bath), Department of Pharmacy and Pharmacology, University of Bath, Bath, BA2 7AY United Kingdom; 2 University College London, Institute of Child Health, London, WC1N 1EH United Kingdom

**Keywords:** Cancer metabolism, Cancer metabolism

## Abstract

To sustain their malignancy, tumour cells acquire several metabolic adaptations such as increased oxygen, glucose, glutamine, and lipids uptake. Other metabolic processes are also enhanced as part of tumour metabolic reprogramming, for example, increased serine metabolism. Serine is a non-essential amino acid that supports several metabolic processes that are crucial for the growth and survival of proliferating cells, including protein, DNA, and glutathione synthesis. Indeed, increased activity of *D*-3-phosphoglycerate dehydrogenase (PHGDH), the enzyme rate-limiting de novo serine synthesis, has been extensively reported in several tumours. Therefore, selective inhibition of PHGDH may represent a new therapeutic strategy for over-expressing PHGDH tumours, owing to its downstream inhibition of essential biomass production such as one-carbon units and nucleotides. This perspective article will discuss the current status of research into small molecular inhibitors against PHGDH in colorectal cancer, breast cancer, and Ewing’s sarcoma. We will summarise recent studies on the development of PHGDH-inhibitors, highlighting their clinical potential as new therapeutics. It also wants to shed a light on some of the key limitations of the use of PHGDH-inhibitors in cancer treatment which are worth taking into account.

## Upregulation of the de novo serine synthesis pathway in cancer cells

Cancer is the second leading cause of death globally, accounting for an estimated 9.6 million deaths of the 17 million cases diagnosed in 2018^1^. Currently, the most commonly employed cancer treatment falls under the umbrella of radiotherapy and chemotherapy. These conventional non-specifically treatments are known for their severe side effects, which warrants further research into specifically targeting cancer cells^2^.

Cancer cells acquire adaptations during their development, known as hallmarks. The hallmarks of cancer, including rapid proliferation, irrepressible metastasis, and the evasion of cell death, are sustained via the reprogramming of cellular metabolic pathways^3^. In recent years, several metabolic vulnerabilities of tumour cells have been extensively investigated for potential therapeutic targets^4^.

New developments within the field of cancer metabolism have shed light on the preferential upregulation of biosynthetic pathways required for tumorigenesis and metastasis^3^. The increased flux through the glycolysis pathway in cancer cells termed the “Warburg effect”, exemplifies cancer metabolism as a fundamental hallmark required to maintain the oncogenic phenotype^3^.

**Fig. 1:**
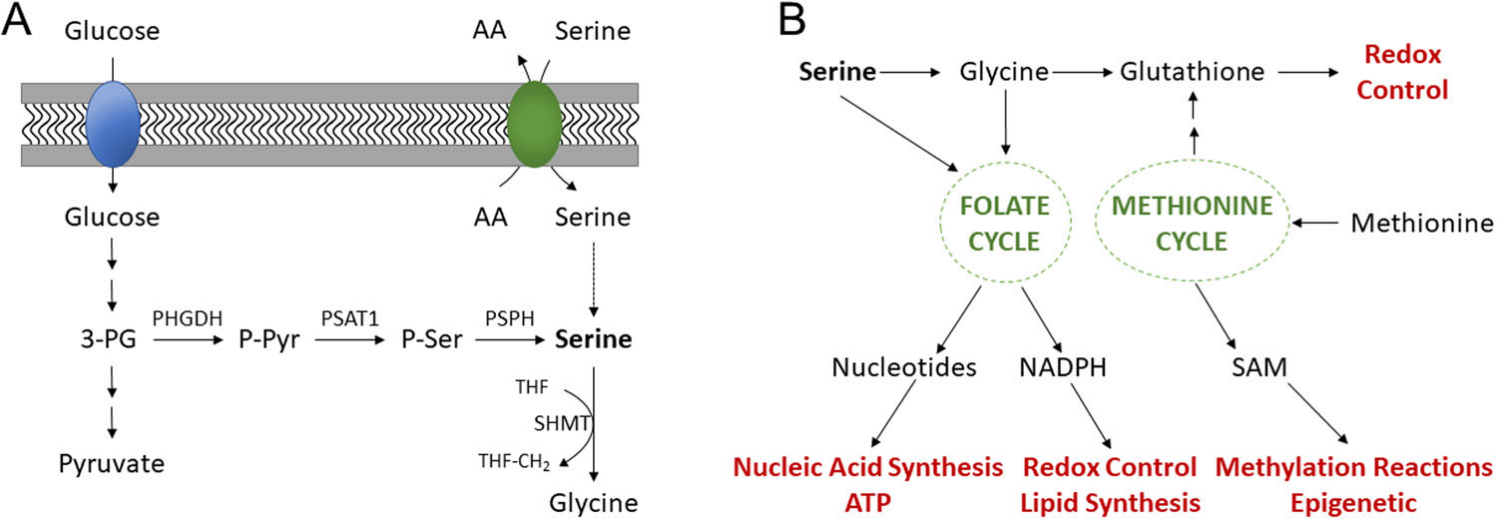
The serine synthesis pathway (SSP) and downstream anabolic pathways. **A** Serine synthesis pathway. Serine is shown to be both imported from the extracellular space by amino acid exchangers and also synthesised de novo from glucose. The first rate-limiting enzyme, PHGDH, oxidises 3-phosphoglycerate (3-PG) using NAD + cofactor to produce phosphohydroxy pyruvate (P-Pyr); secondly, phosphoserine aminotransferase (PSAT1) transaminates P-Pyr to phosphoserine (P-Ser) using glutamate (Glu) as the nitrogen donor; lastly, phosphoserine phosphatase (PSPH) hydrolyses P-Ser to serine. Serine is directly incorporated into lipid head groups and proteins. Serine-derived glycine and folate one-carbon units contribute to nucleotide synthesis [adapted from 12]. **B** The role of serine in downstream anabolic pathways. Serine is a crucial contributor of one carbon units feeding into one carbon metabolism, the folate cycle and the methionine cycle. One-carbon units donated as a result of serine metabolism are used in the anabolic synthesis of cellular building blocks, co-factors and reducing factors. This figure shows the downstream anabolic pathways which use serine derivatives, and the cellular functions (red) which they support, such as biomolecule synthesis, maintenance of cellular homoeostasis, redox reactions and post-translational modifications.

Serine is a conditionally essential amino acid, and a central precursor for biosynthetic metabolism^5^. The de novo serine synthesis pathway (SSP) forms one branch of glucose metabolism, utilising the glycolic intermediate 3-phosphoglycerate (3PG) for the synthesis of serine (Fig. 1A). The SSP has been implicated as a critical pathway enabling tumour growth and proliferation, as serine is an essential source of one-carbon units fundamental for nucleotide synthesis^6^.

The significance of serine for tumorigenesis has been acknowledged, with oncogenic cells obtaining serine both exogenously via diet, and the SSP, to satisfy their requirement for rapid proliferation^6^. One-carbon metabolism and autophagy, a process that removes damaged and unnecessary cell parts, have both been implicated in cancer progression, with a link between the two recently identified. Emerging studies have shown autophagy-derived serine feeds directly into one-carbon metabolism, further supporting protein synthesis and adjustment to respiratory growth^7^. Indeed, the inhibition of serine metabolism has been extensively investigated and has the potential to treat different cancers including melanomas, cervical cancer, and colorectal cancer^8–10^.

## PHGDH enzyme: a novel target for cancer treatment

**Fig. 2:**
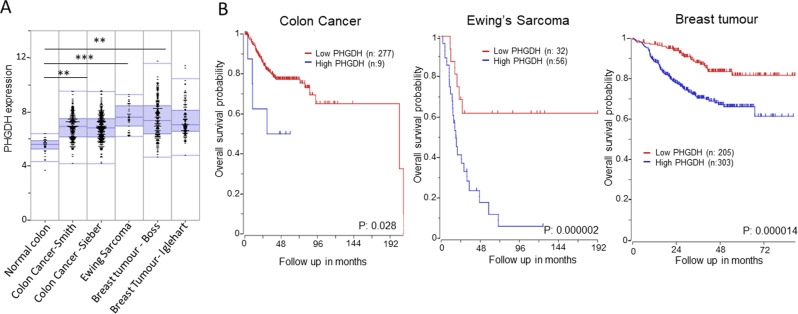
PHGDH expression is a poor prognostic marker for colon cancer, Ewing’s sarcoma and breast tumours. **A** Analysis of PHGDH expression levels in normal colon (32 patients-Marra), colon cancer (232 and 290 patients-Sieber), Ewing’s Sarcoma (37 patients-Francesconi) and breast tumours (204 and 123 patients-Bos). (**P:0.0025; ***P:0.0001) **B** Kaplan-Meier survival curve based on high and low PHGDH expression levels in colon cancer, Ewing’s sarcoma and breast tumours derived from SieverSmith, Savola and Booser cohort with 355, 117 and 508 patients, respectively. Tumours with high PHGDH expression showed decreased survival. Data were acquired from the R2:GenomicsAnalysisandVisualizationPlatform and significance is calculated with a one-way ANOVA between groups.

The serine synthesis pathway has been widely reported to be strongly associated with cancer proliferation and metastasis, although the specific mechanism underpinning the functional addiction of some tumour cells to serine remains uncertain^11^. Serine is widely recognised as an essential component for anabolic processes on which rapid proliferation is dependent^11^. Therefore, upregulation of serine metabolism is frequently observed in tumour cells. PHGDH has been demonstrated to be a critical enzyme in serine biosynthesis and is overexpressed in multiple tumours^7^. Analysis of published data sets shows upregulation of PHGDH expression in colon cancer, Ewing’s sarcoma and breast cancer (Fig. 2A). This, in conjunction with a significant correlation between high PHGDH expression and poor patient survival in these cancers, may make PHGDH inhibition an attractive therapeutic strategy for these tumours (Fig. 2B).

### Colorectal cancer (CRC)

Serine has been identified as an essential component in sustaining CRC cell growth, through the synthesis of purine and one-carbon units^12^. In support of this, CRC cells have been shown to exhibit functional addiction to serine in xenograft models^13^. Further, serine was observed to maintain the methionine cycle and *S*-adenosyl-methionine production with stable-isotope tracing such as ^13^C serine (Fig. 1B)^12^.

When comparing CRC versus non-tumour tissue with tissue microarray and immunohistochemistry, PHGDH mRNA and protein levels have been identified as upregulated, suggesting it to be a tumour-specific target^10^. In CRC patients, elevated PHGDH protein expression strongly correlated to the poor clinical outcome (Fig. 2A and 2B), further suggesting a key role for PHGDH in promoting CRC progression and metastasis^10^.

### Ewing’s sarcoma (EWS)

EWS is a bone tumour driven by a translocation event, resulting in the EWS/FLI1 (EF) fusion protein^14^. EF acts as an oncogenic transcription factor, which modifies the expression of thousands of genes favouring tumorigenesis, one of which being *PHGDH*^14^. Tanner et al. reported higher PHGDH protein expression in EWS tumour tissue than adjacent normal tissue^14^.

Importantly, high PHGDH expression shows a strong correlation with worse patient survival (Fig. 2A, B). This correlation is significant, as it confirms critical clinical implications of the EF-driven upregulation of PHGDH^15^. Further, Tanner et al. show that PHGDH knockdown and pharmacological inhibition with NCT-502 and NCT-503, in vitro impaired EWS cell proliferation and induced cell death^15^.

In the same year, Svoboda et al. demostrated EWS dependency on serine metabolism in vivo. PHGDH inhibition and knockdown both resulted in suppression of cell proliferation and viability, in a xenograft mouse model^16^. Interestingly, these observations were reported on cells culture with high concentrations of serine/glycine. This finding demonstrated that EWS cells have a preference for serine via PHGDH and the SSP, with an abundance of exogenous serine in culture media unable to rescue the loss of PHGDH function^16^. The deleterious effect of PHGDH knockdown irrespective of the availability of exogenous serine strongly supports the case for clinical development into a selective PHGDH inhibitor.

### Breast cancer

High PHGDH expression has been identified in multiple subsets of breast cancer (Fig. 2A, B), such as triple-negative, and oestrogen receptor (ER) negative breast cancer^17^. Possemato et al., Locasale et al., and Unterlass et al. reported that PHGDH depletion suppressed ER-negative breast cancer cells using an shRNA against PHGDH in an in vivo model^7,9,18^. Notably, overexpression of PHGDH in non-malignant breast epithelial cells induces an increase in cell proliferation associated with the alteration in cellular serine metabolism observed in tumorigenesis^8^. These discoveries were used to evidence the claim that *PHGDH* is a fundamental requirement for the tumorigenesis of ER-negative breast cancer cells^18^.

Taken together these results show the central role of PHGDH in maintaining the oncogenic phenotype in CRC, EWS, and breast cancer.

## Potential therapeutic approaches

It is clear that inhibition of cancer proliferation with PHGDH inhibitors is a promising therapeutic avenue. Unfortunately, there are currently no drugs targeting PHGDH approved by the Food and Drug Administration (FDA), despite reports of several small molecule inhibitors (SMI)^19^. Several experimental inhibitors are currently undergoing optimisation with the hope of clinical trials in the near future^19^. Through high-throughput screening, Mullarky et al. identified a PHGDH SMI known as CBR-5884, in a library of 800,000 compounds^19^. Using an in vitro PHGDH enzyme assay, they determined CBR-5884 to be a weak and selective PHGDH inhibitor (IC_50_ = 33 ± 12 mM), whose mode of action involves the disruption of the enzymes tetrameric state in favour of the dimeric conformation^20^. This discovery has provided insights into the functionality and 3D conformations of PHGDH in its active state. Importantly, this suggests that its activity in situ may be regulated by fluctuations between dimeric and tetrameric conformations^20^. Furthermore, using an analogous assay, Pacold et al. identified a compound named NCT-503 to elicit an improved inhibition of PHGDH (IC_50_ = 2.5 ± 0.6 mM) (Fig. 2F)^21^. Both CBR-5884 and NCT-503 have performed consistently experimentally showing promising anti-cancer activity in vitro. To their detriment, they suffer from limited potency against PHGDH in vivo^20,21^. They do, however, demonstrate this approach to be a viable strategy to be pursued as a novel oncology therapeutic avenue. These compounds are currently undergoing optimisation for future clinical testing based on their success in a research-based environment.

Perhaps the most exciting PHGDH SMI development is the discovery of compound X by AstraZeneca. Through an innovative fragment-based lead generation screening, they identified compound X to covalently bind to the adenine region of the NAD^+^ cofactor binding pocket with a sub-micromolar affinity (*K*_D_ = 0.18 µM)^22^. Unfortunately, pre-clinical data of compound X is not yet available. During 2017 and 2018, a surge occurred in the disclosure of patent applications for indole amide-based PHGDH SMI by Raze Therapeutics and Boehringer Ingelheim, respectively^23^. Pre-clinical data for these compounds is not yet disclosed. Most recently, a series of indole amide compounds have been developed which, in enzymatic assays, inhibited PHGDH with potencies lying within the low nanomolar range, markedly lower than any previously reported^23^. These indole amide compounds were also reported to bind within the adenine site, however in a non-covalent manner^23^. Taken together, these data suggest that there is an active pre-clinical pipeline line of research of PHGDH inhibitors undergoing development as potential novel oncology therapeutics.

## Limitations and considerations

Despite the promise PHGDH SMI provides to patients, there are important considerations to take into account. SMI targeting cancer metabolism has the potential to elicit detrimental side effects on normal cells, as observed with anti-glycolytics such as lonidamine^24^. Therefore, attention must be paid to the limitations of this strategy. As observed with SMI such as vemurafenib in melanomas, development of resistance has been observed, and is recognised to be a potential limitation of SMI^25^. With the ubiquitous expression of proteins and low tissue selectivity, particular attention must be paid to the potential blanket effect of inhibiting PHGDH. This side-effect must be pre-empted and, through optimisation, potentially circumvented. Moreover, the issue of inhibiting serine production in wild-type cells must be addressed, as this conditionally essential amino acid is required for normal cellular function. Apropos to this, increasing exogenous serine has been demonstrated to ameliorate serine deficiency disorders containing hypomorphic PHGDH mutations^26^. This suggests exogenous serine supplements an adjuvant to PHGDH inhibition may sustain normal, but not oncogenic, cellular function. Studies have also reported tumour cells to favour serine de novo, on account of its in situ production easing accessibility, and resultantly minimising dietary dependence^16^. This finding is promising, suggesting normal cell function can be kept buoyant through monitoring dietary serine levels. In addition, the pre-clinical use of NCT-503 has identified it to arrest embryonic development within the central nervous system (CNS) in mouse models, as a result of its permeability to the blood-brain barrier (BBB)^27^. Therefore, to evade CNS off-target effects, it is desirable to develop SMI with elevated lipophilicity to prevent the inhibitor from entering into the brain. In conjunction with dietary serine supplements and existing treatment methods such as chemotherapy or radiotherapy, this therapy may be safe in teenagers and non-pregnant adults. There is scientific precedence for the success of such combination therapies, as it reduces the dosage of both the chemotherapy/radiotherapy and the SMI, minimising any potential side effects^28^. Most recently, nanoparticles are emerging as an attractive targeted delivery system to reduce the systemic toxicity of anti-cancer therapeutics. Currently, carboxymethyl chitosan is the leading choice of nanomaterial for anti-cancer therapy delivery due to its favourable solubility, comparability, and biodegradability properties^29^. Such a targeted delivery system would avoid the emergence of off-target effects in the CNS, but also reduce hepatic and renal toxicity normally observed with anti-tumour agents. In support of modifying delivery and release of anti-cancer treatments, application of site-specific delivery for cisplatin has been deemed successful, leading to reduced systemic toxicity and prologued release^29^. Despite these challenges, by exploiting novel approaches, new hope may be brought to patients suffering from the burden of a cancer diagnosis.

## Conclusions and perspectives

To target the metabolic vulnerabilities of cancer is not a task undertaken lightly, as with it comes the grave risk of impeding the function of normal cells. In the development of novel oncology therapeutics, it must be taken into consideration that, by default, most enzymes employed in tumorous metabolism also play key roles in normal cells. The significance of this consideration has been exemplified by the subset of therapeutics developed to target glycolysis, such as lonidamine, whose clinical success has been arrested due to significant pancreatic and hepatic toxicity^24^. Due to the ubiquitous nature of metabolic enzymes, a better understanding of cancer metabolism is required to circumvent the systemic toxicity observed with anti-glycolytic therapeutics.

As a group of diseases, cancer is an undiscriminating and serious threat to human life. A growing subset of cancers are being identified to shunt glycolytic intermediates into the SSP, generating serine de novo, and facilitating the synthesis of nucleotides and other essential biomass (Fig. 1B). Thus, based on the evidence presented in this review, PHGDH is not only a viable, but an exciting therapeutic target to block the rapid cell proliferation and metastasis characteristic of cancer cells. Whilst the complete role of PHGDH in all cancers, and the implications of inhibiting it, have not yet been determined, its therapeutic potential is promising. Moreover, combinatorial approaches utilising chemotherapy, radiotherapy, and dietary serine supplements could be considered in the short term, with long-term hopes resting on the shoulders of targeted and site-specific delivery systems. On balance, the future pipeline targeting cancer metabolism, with specific regard to inhibiting PHGDH, looks hopeful in ameliorating cancer metastasis and disease progression.
